# Predictive models demonstrate age‐dependent association of subcortical volumes and cognitive measures

**DOI:** 10.1002/hbm.26100

**Published:** 2022-10-12

**Authors:** Akila Weerasekera, Adrian Ion‐Mărgineanu, Christopher Green, Maria Mody, Garry P. Nolan

**Affiliations:** ^1^ Department of Radiology, Athinoula A. Martinos Center for Biomedical Imaging Massachusetts General Hospital, Harvard Medical School Boston Massachusetts USA; ^2^ ESAT – STADIUS KU Leuven Leuven Belgium; ^3^ Biomed Artificial Intelligence Bucharest Romania; ^4^ Department of Diagnostic Radiology Detroit Medical Center & Wayne State School of Medicine Detroit Michigan USA; ^5^ Department of Microbiology & Immunology Stanford University School of Medicine Palo Alto California USA

**Keywords:** aging, cognitive skills, intelligence, magnetic resonance imaging, random forest classifiers, subcortical brain volumes

## Abstract

Whether brain matter volume is correlated with cognitive functioning and higher intelligence is controversial. We explored this relationship by analysis of data collected on 193 healthy young and older adults through the “Leipzig Study for Mind–Body–Emotion Interactions” (LEMON) study. Our analysis involved four cognitive measures: fluid intelligence, crystallized intelligence, cognitive flexibility, and working memory. Brain subregion volumes were determined by magnetic resonance imaging. We normalized each subregion volume to the estimated total intracranial volume and conducted training simulations to compare the predictive power of normalized volumes of large regions of the brain (i.e., gray matter, cortical white matter, and cerebrospinal fluid), normalized subcortical volumes, and combined normalized volumes of large brain regions and normalized subcortical volumes. Statistical tests showed significant differences in the performance accuracy and feature importance of the subregion volumes in predicting cognitive skills for young and older adults. Random forest feature selection analysis showed that cortical white matter was the key feature in predicting fluid intelligence in both young and older adults. In young adults, crystallized intelligence was best predicted by caudate nucleus, thalamus, pallidum, and nucleus accumbens volumes, whereas putamen, amygdala, nucleus accumbens, and hippocampus volumes were selected for older adults. Cognitive flexibility was best predicted by the caudate, nucleus accumbens, and hippocampus in young adults and caudate and amygdala in older adults. Finally, working memory was best predicted by the putamen, pallidum, and nucleus accumbens in the younger group, whereas amygdala and hippocampus volumes were predictive in the older group. Thus, machine learning predictive models demonstrated an age‐dependent association between subcortical volumes and cognitive measures. These approaches may be useful in predicting the likelihood of age‐related cognitive decline and in testing of approaches for targeted improvement of cognitive functioning in older adults.

## Graphical abstract

Whether brain matter volume, as determined by magnetic resonance imaging, is correlated with cognitive functioning and higher intelligence is controversial. We explored this relationship by analysis of data collected on 193 healthy young and older adults through the “Leipzig Study for Mind–Body–Emotion Interactions” study. Random forest feature selection analysis showed that cortical white matter was the key feature in predicting fluid intelligence in both young and older adults; however, crystallized intelligence, cognitive flexibility, and working memory were best predicted by the volumes of different sub‐regions of the brain in younger and older groups. Thus, machine learning‐based predictive models demonstrated an age‐dependent association between subcortical volumes and cognitive measures. These approaches may be useful in predicting the likelihood of age‐related cognitive decline and in testing approaches for targeted improvement of cognitive functioning in older adults.

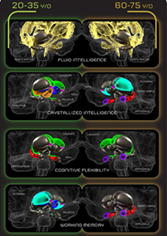

## 1 INTRODUCTION

Whether brain matter volume is correlated with cognitive functioning and higher intelligence is a much discussed, frequently researched topic in neuroscience that remains controversial. Connectivity analysis performed using structural and functional magnetic resonance imaging (MRI) has shown an association between intelligence and putamen (Ketteler et al., 2008; Mestres‐Missé et al., 2010), caudate (Basten et al., 2015; Grazioplene et al., 2015; Rhein et al., 2014), hippocampal (Valdés Hernández et al., 2017), and thalamic (Bohlken et al., 2014) volumes. MRI studies suggest that neurobiological correlates of intelligence are configured by specific neural networks connecting cortical and subcortical brain structures (Basten et al., 2015; Burgaleta et al., 2014; Cacciola et al., 2017; Cox et al., 2019). For example, the prefrontal cortex (Courtney et al., 1998; Kane & Engle, 2002), which is regarded as the processing center for higher cognitive functions, shows strong connectivity with the subcortical brain regions, specifically the basal ganglia‐striatal region, which is a region classically associated with motor processes (Abdullaev et al., 1998; Middleton & Strick, 2000). Frontal‐subcortical circuitry supports important executive functions, and there is evidence that subcortical structures have independent roles in higher cognition (Crosson, 2021; Jung et al., 2020; Leisman et al., 2014; Moretti et al., 2017). A recent study showed that the subcortical structures, such as the nucleus accumbens and amygdala (linked to reward processing in judgment, decision‐making, and emotion regulation and previously thought to have minimal association with intelligence), have a strong connection to hippocampal morphometry, a structure strongly associated with general intelligence (Wu et al., 2020). General intelligence encompasses fluid and crystallized intelligences. Fluid intelligence involves comprehension, reasoning, and problem solving, whereas crystallized Intelligence involves retrieval of stored information and past experiences. Both fluid intelligence and crystallized intelligence have strong associations with subcortical volumes and subcortical connectivity with prefrontal regions (Colom et al., 2009; McGrew, 2009).

Further evidence in support of subcortical involvement in cognitive performance comes from studies of neuropsychiatric disease. In subjects with autism spectrum disorder, the volumes of the caudate, putamen, and amygdala are usually increased relative to neurotypical controls. The hyperconnectivity of these structures to frontal regions in those with autism spectrum disorder may account for traits such as repetitive, stereotyped behaviors, impaired executive control, and poor social skills (Qiu et al., 2016; Sato et al., 2014; Stanfield et al., 2008). A study employing children who survived preterm birth without major disability showed that lower intelligence quotients (IQs) are related to poorer development of the caudate relative to other brain structures (Abernethy et al., 2004). In another example, Chand et al. using structural MRI images and machine learning algorithms discovered two distinct neuroanatomical subtypes of schizophrenia. Compared to normal participants, subjects with subtype 1 had smaller thalamus and nucleus accumbens volumes, and subjects with subtype 2 had increased volume of the basal ganglia. Furthermore, they reported higher educational attainment in those with subtype 2 compared to subtype 1, which may indicate a role of the basal ganglia in higher cognitive achievement (Chand et al., 2020). Another recent study showed that electrical stimulation of the caudate and putamen improved learning and memory ability in epileptic patients, further suggesting the importance of striatal structures in higher‐order cognitive functioning (Bick et al., 2019).

A number of neuroimaging studies have also reported age‐related volume changes of various subcortical structures. For the most part, these studies have found volume reductions with age (Du et al., 2006; Hasan et al., 2008; Sullivan et al., 2004; Van Der Werf et al., 2001; Walhovd, Fjell, Reinvang, Lundervold, Dale, et al., 2005), although others have found age‐related increases in volume in some structures (Goodro et al., 2012; Van Petten, 2004). Given the current demographic trends reflecting an aging society and an increasing incidence and burden of neurodegenerative diseases such as Alzheimer's disease and Parkinson's disease (Nichols et al., 2019), a closer look at subcortical changes in age‐related cognitive decline may help shed light on the etiologies of these conditions, which remain largely unknown.

Using advanced artificial intelligence methods informed by neuroimaging metrics of healthy aging, early detection of abnormal brain changes due to disease may be possible. Machine learning and deep learning studies have demonstrated associations between the subcortical volumes and cognitive performance. In a pioneering study, Wang et al. (2015) showed that IQ could be predicted in developing children using support vector regressors; predictive features were thalamus, hippocampus, amygdala, and caudate volumes. Using these features, Zou et al. (2019) analyzed a much larger data set with a deep learning approach and showed that of these three regions, thalamus and the caudate nucleus volumes were most predictive of IQ in developing children.

To understand the relationship between subcortical volumes and higher‐level cognition in a nonclinical sample, we conducted a study with the open‐access “Leipzig Study for Mind–Body–Emotion Interactions” (LEMON) database of MRI and cognitive data from 193 subjects (Babayan et al., 2019). Random forest is the best off‐the‐shelf algorithm for a large number of real‐world classification problems (Fernández‐Delgado et al., 2014). Although it has been a central topic of interest for the last two decades in machine learning communities, the exact statistical foundations are still being researched (Mentch & Zhou, 2020). In short, the random forest algorithm is based on the aggregation of a large number of decision and regression trees, where each tree is trained independently on a randomly selected subset of data points from the original data set and on a randomly selected subset of features. At each node in the tree there will be a split and the algorithm will select the threshold for a feature that maximizes the information gain of that split. This is applied until there are no more points in the tree's data set. Since each tree is trained independently from each other, the risk of overfitting when using a large number of trees is very low (Fernández‐Delgado et al., 2014; Probst & Boulesteix, 2018), and it is now recommended to use as many trees as one possibly can (Mentch & Zhou, 2020) In addition, the implicit randomization in random forests acts as regularization and mitigates against overfitting to particular features (Mentch & Zhou, 2020). It has also been shown that random forests adapt very well to sparsity, thus its convergence depends only on the number of strong features and not on how many noise variables are present (Biau, 2012).

Given the moderate number of subjects in our data set, we used a fivefold cross‐validation scheme stratified by gender for a group of young adults and a group of older adults, because we were interested in predictions corresponding to all subjects, not just a random validation subset. In contrast, the winning group of the Adolescent Brain Cognitive Development Neurocognitive Prediction (ABCD‐NP) Challenge used a random 20% validation set (Mihalik et al., 2019). To measure the confidence of comparing the predictive power of subcortical volumes to that of global brain volumes, we ran 1000 cross‐validation simulation studies, so that we averaged out the randomness induced by the cross‐validation train‐test split and the random internal splits. We thereby computed the true difference between predicting a cognitive measure based on subcortical volumes compared to global brain volumes and extracted a hierarchy of the most predictive brain subregions for the selected cognitive functions. Given the role of subcortical structures in cognition and age‐related changes in brain structures, we expected subcortical structures to predict differences in cognitive measures between young versus older adults. Indeed, we found that cortical white matter was the key feature in predicting fluid intelligence in both young and older adults. Crystallized intelligence, cognitive flexibility, and working memory were best predicted by subcortical volumes in both younger and older groups. Discovering brain structure and cognitive associations specific to aging is expected to be vital for understanding the neural underpinnings of cognition and cognitive decline and for identification of biomarkers of neurological disorders.

## 2 MATERIALS AND METHODS

### 2.1 Study subjects

The open‐access LEMON data set includes extensive clinical and cognitive information and raw anonymized MRI scans of subjects grouped in age bins of 5 years (e.g., 20–25 and 25–30 years of age) (Babayan et al., 2019). Subjects were evaluated at the Max Planck Institute for Human Cognitive and Brain Sciences. Exclusion criteria included current or past history of cardiovascular disease (hypertension, congenital heart disease, or heart attacks), psychiatric disorders needing more than 2 weeks of therapy during the last 10 years, post‐traumatic stress disorder, psychosis or suicidal attempts, neurological (e.g., multiple sclerosis, epilepsy, and stroke), or malignant conditions, and medication usage (e.g., centrally acting drugs, cortisol, alpha/beta‐blocker, excessive alcohol, benzodiazepine, cocaine, amphetamines, cannabis, or opiates) as well as MRI contraindications (Babayan et al., 2019; Mehrabinejad et al., 2021). A total of 224 eligible native German‐speaking individuals underwent structural MRI scans, psychological assessments, and cognitive‐, attention‐, and creativity‐related assessments (Mehrabinejad et al., 2021). Magnetic resonance imaging was performed on a 3 T scanner (MAGNETOM Verio, Siemens Healthcare GmbH) equipped with a 32‐channel head coil. Other technical details of protocols are available at http://fcon_1000.projects.nitrc.org/indi/retro/MPI_LEMON.html.

Of the 224 subjects, we selected a subset of 193 healthy subjects who had completed all cognitive testing without any issues. Some of the encountered issues included forgetting their reading glasses, being distracted or not understanding the instructions, having set the wrong difficulty level, technical errors during test performance, and intentionally not starting the test on time. Furthermore, we used only those age groups that had a minimum of 10 subjects. Our cohort included 68 females and 125 males from six age groups, three groups representing young adults (YAs: 20–35 years) and three groups for older adults (OAs: 60–75 years). Since there was a significant gender difference between the young and older age groups (*χ* ^2^(1) = 4.91, *p* = .026), we used a gender‐stratification cross‐validation scheme to mitigate overfitting of the data.

### 2.2 Cognitive tests

Cognitive skills were tested in four domains: fluid intelligence, crystallized intelligence, cognitive flexibility, and working memory. (a) Performance on subtest 3 of the *Performance Testing System* (Kreuzpointner, 2010), a measure of logical thinking, was used to quantify fluid intelligence. Participants were asked to identify one item in a series of symbols that did not follow the logical rule of that series. The goal was to find as many items as possible within 3 min. The number of correctly identified items was used as the measure of fluid intelligence. (b) *The Vocabulary Test (Wortschatztest)* (Schmidt & Metzler, 1992), a measure of receptive vocabulary and considered an index of verbal IQ, was used to measure crystallized intelligence. (c) *The Trail Making Test* (Tischler & Petermann, 2010), a measure of cognitive flexibility, was used to measure cognitive processing speed and executive function. In the second subtest, the subject connects numbers and letters in alternating and increasing order. The completion time was taken as our measure of cognitive flexibility. (d) *The Test of Attentional Performance (Working memory task)* (Zimmermann & Fimm, 2017) was used to quantify working memory. The participants had to monitor a series of numbers and press a button as fast as possible when the number on the screen was the same as the number presented two trials earlier. The mean reaction time for correct button presses was used as the measure. The performance of the cohort used in this study on these cognitive measures arranged by age group and gender can be found in Table 1.

**TABLE 1:** Subject characteristics and performance on cognitive measures^a^

	Age groups (years)										
	Young adults (20–35 years)			Older adults (60–75 years)			Young versus older		Genders combined		
	Female	Male	*p*	Female	Male	*p*	*p*_*Female*_	*p*_male_	*Young*	*Older*	*p*
*N*	44	101	–	23	25	–	.027^b^				
FI (items)	22.2 (3.6)	20.8 (3.3)	.030	15.9 (3.1)	15.9 (3.1)	1	<.001	<.001	21.2 (3.4)	15.9 (3.0)	<.0001
CI (IQ scores)	109.0 (8.5)	107.3 (8.4)	.270	107.4 (9.6)	109.6 (12.3)	.491	.505	.383	107.8 (8.4)	108.6 (11)	.5980
CF (s)	47.5 (12.9)	52.8 (15.7)	.036	84.4 (27.8)	95.5 (32.2)	.207	<.001	<.001	51.1 (15.0)	90.1 (30.0)	<.0001
WM (ms)	560.0 (163.2)	553.7 (135.9)	.823	641.8 (194.2)	589.0 (133.5)	.283	.092	.244	555.3 (143.3)	614.3 (164.0)	.0187

*Note*: CI is reported here as standardized scores, scores on the rest are raw score averages.

Abbreviations: CF, cognitive flexibility; CI, crystallized intelligence; FI, fluid intelligence; WM, working memory.

^a^ Data are reported as averages (standard deviation).

^b^ Sex differences between groups (young vs. older adults) were compared using chi‐squared test.

### 2.3 Brain segmentation

T1‐MPRAGE images were processed and segmented using Free Surfer v5.3.0 (Desikan et al., 2006). Processing steps included motion correction, skull‐stripping, Talairach transformation, signal‐intensity normalization, subcortical processing, and volumetric segmentation. All segmentation results were visually inspected for quality before inclusion in the analyses. Segmented volumes of each subcortical structure (caudate, putamen, thalamus, pallidum, amygdala, nucleus accumbens, and hippocampus) for both hemispheres were extracted and summed. To calculate global brain volume, volumes of total gray matter, cortical white matter for both hemispheres, and cerebrospinal fluid (CSF) were summed. Volumes of all brain segments were normalized to the estimated total intracranial volume (eTIV) to control for individual and sex‐related differences in brain size as previously described (O'Dwyer et al., 2012; Wolff et al., 2013). Detailed volumetrics of the brain subregions categorized by subject age group and gender are listed in Table 2.

**TABLE 2:** Brain subregion volumes (in mm^3^) of young and older adults in our cohort^a^

	Age groups (years)					
	Young adults (20–35)		Older adults (60–75)		Young versus older^b^	
	Female (*N* = 44)	Male (*N* = 101)	Female (*N* = 23)	Male (*N* = 25)	*p*_*Female*_	*p*_male_
eTIV	1,047,425 (84,677)	1,170,756 (101,342)	1,024,260 (117,260)	1,170,432 (137,151)	.407	.991
tGMV	620,627 (48,493)	694,296 (55,759)	535,971 (32,449)	584,346 (51,651)	<.001	<.001
cWM	423,257 (33,411)	495,464 (50,506)	415,921 (41,849)	460,488 (60,589)	.471	.012
CSF	958 (216)	1049 (239)	1241 (278)	1537 (320)	<.001	<.001
Caudate	7582 (892)	8095 (1073)	7212 (874)	7466 (762)	.109	.001
Putamen	11,189 (983)	12,442 (1260)	9296 (1061)	10,284 (1066)	<.001	<.001
Thalamus	14,015 (1028)	15,814 (1253)	12,288 (939)	13,076 (1481)	<.001	<.001
Pallidum	2918 (346)	3305 (401)	2857 (394)	3169 (381)	.534	.122
Amygdala	2816 (262)	3263 (434)	2705 (291)	2971 (413)	.133	.003
NAcc	1137 (134)	1293 (189)	903 (171)	927 (167)	<.001	<.001
Hippocampus	6870 (603)	7623 (698)	6834 (558)	6838 (846)	.808	<.001

Abbreviations: CSF, cerebrospinal fluid; cWM, combined white matter volume; eTIV, estimated total intracranial volume; NAcc, nucleus accumbens; tGMV, total gray matter volume.

^a^ Data are reported as averages (standard deviation).

^b^ *p* Values are reported: young females versus older females (*p*_female_) and young males versus older males (*p*_male_).

### 2.4 Simulations

**FIGURE 1:**
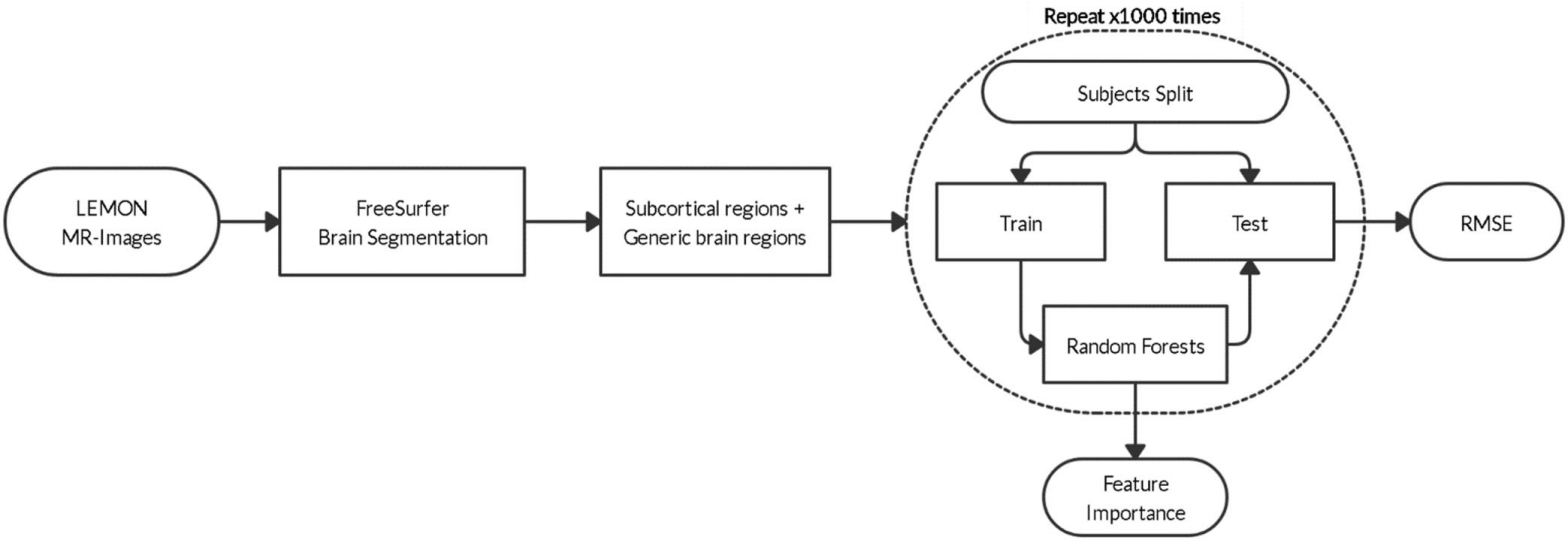
Schematic illustration of the overall workflow

All software code and processed data sets are available upon reasonable request. The simplified workflow is presented in Figure 1. The volumes of three large regions of the brain (gray matter, cortical white matter, and CSF) normalized to eTIV and seven eTIV‐normalized subcortical volumes were used as quantitative descriptors, which we refer to here as features, in simulations. To properly analyze the predictive power of subcortical volumes, a three‐way comparison was done using the volumes of the larger regions as baseline, the subcortical volumes as the main set of interest, and the union of the two (global brain volumes and subcortical volumes) as the combined data set. A thousand simulations of cross‐validation training of the random forest algorithm with 1000 regression trees were performed in Python using the scikit‐learn package (Pedregosa et al., 2011). Nonparametric tests included in Python's “SciPy” (Virtanen et al., 2020) and “scikit‐posthocs” (Terpilowski, 2019) packages were used to determine statistical significance. In each simulation, all subjects from the young adult and older adult age groups were randomly split into five groups stratified by gender, as the data set is unbalanced toward young males (Table 1). The algorithm was trained five different times, with each training iteration done on a training set consisting of four folds, with the remainder used as the test set. The stratification by gender ensured that the gender distribution of the test set was as close as possible to that of the training set. Simulations were repeated 1000 times, and significance was evaluated using a nonparametric Kruskal‐Wallis (Kruskal & Wallis, 1952) test and a post hoc Dunn–Šidák test (Grazioplene et al., 2015; Šidák, 1967). These simulations allowed us to compute the true difference between the three data sets in predicting a cognitive measure.

### 2.5 Performance measures

We used root mean squared error (RMSE) as the primary measure to quantify the prediction performance of the random forest algorithm trained on different data sets. Five different RMSEs corresponding to each cross‐validation were computed in each simulation, and the average of these five RMSEs was representative of one simulation. For each of the four cognitive measures, we obtained three RMSE sets, corresponding to each of the three data sets (global volumes, subcortical volumes, and combined), each RMSE set containing 1000 average RMSEs following 1000 simulations. We used the nonparametric Kruskal–Wallis statistical test to determine if the three RMSE sets followed the same distribution or not. A Dunn–Šídák post hoc test was used to identify the RMSE set with the lowest error. We show boxplots of RMSE values for each cognitive measure as well as the *p* values of the data set comparison in each group (YA and OA) with regard to each cognitive variable of interest. Coefficients of correlation between cognitive measures and brain subregion volumes were compared between younger and older adults after converting into *z*‐scores (Fisher, 1921).

### 2.6 Feature importance

One advantage of using the random forest algorithm is that it automatically computes the selection importance of each feature, in our case brain subregion volumes. The feature importance is normalized per data set, so that all values sum to 1. Random forest feature ranking has an internal bias toward the categorical feature with the most categories (Strobl et al., 2007). As our three data sets contain only numerical features, we can reliably use the feature selection rankings from random forest analysis to determine the hierarchy of the most informative brain subregions for each data set. Within each simulation, five different brain subregion rankings were computed corresponding to each of the five folds. We analyzed the brain subregion importance rankings by comparing the average of 1000 simulations to the value that would be obtained if all features had equal importance. The equal importance threshold for the global volume data set of the three large regions of the brain is 1/3, that for the subcortical volumes data set is 1/7, and that for the union of generic and subcortical volumes is 1/10. We performed the comparison using a one‐sample *t*‐test, which is a test for the null hypothesis that the expected mean of a sample of independent observations (i.e., set of feature importance) is equal to the mean of a given population (i.e., theoretical value).

## 3 RESULTS

**FIGURE 2:**
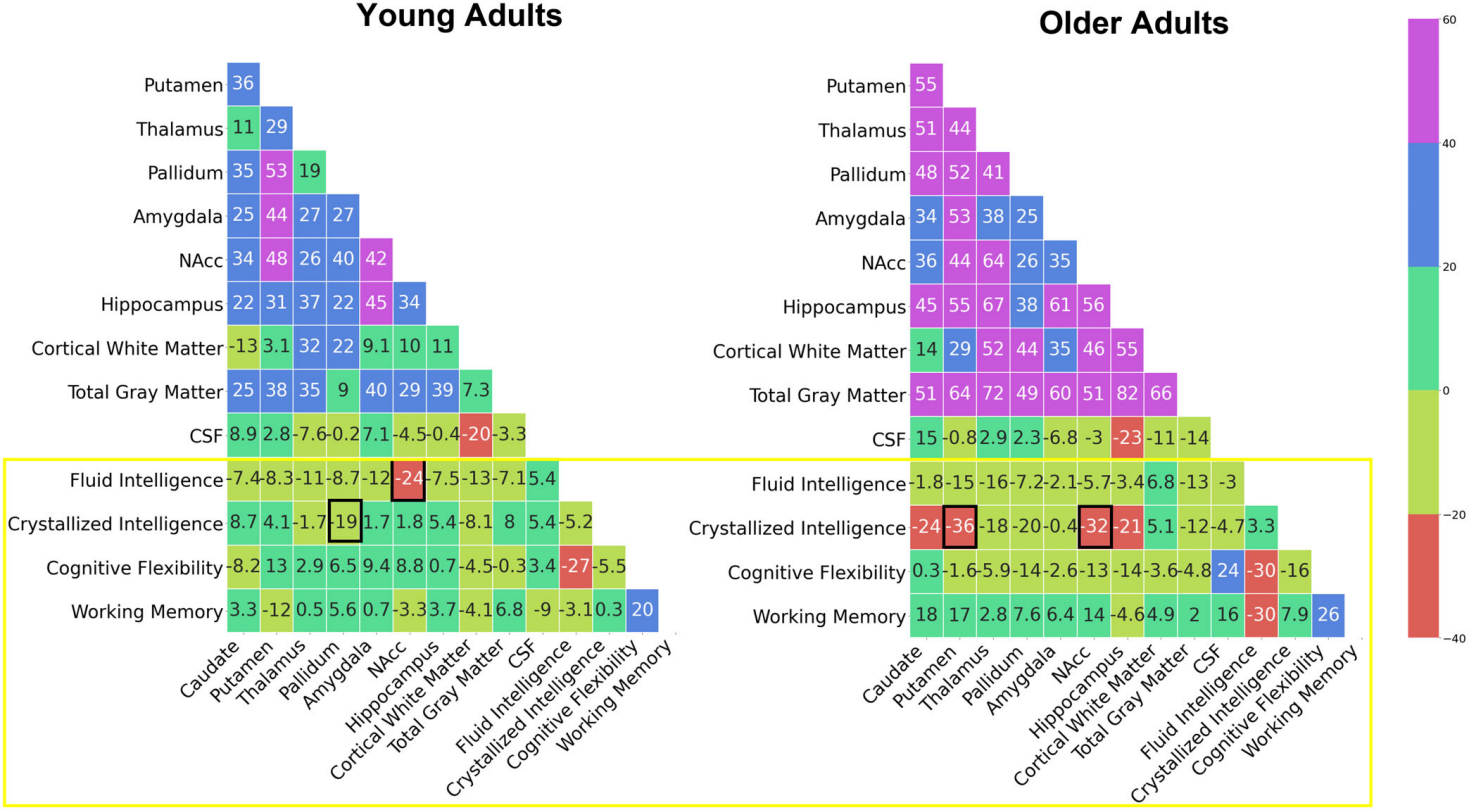
Pearson's correlation coefficient matrices of the variables used in the study. The variables of interest, subcortical volumes, and cognitive measures are highlighted by the yellow box, significant correlations (*p* < .05) within group are highlighted by black boxes. Color scale shows the correlation coefficient values. Correlation coefficients were multiplied by 100 for clarity. CSF, cerebrospinal fluid; NAcc, nucleus accumbens.

In this study, we investigated the association between cognitive functioning and volumes of subcortical regions in a cohort of 193 healthy young (20–35 years) and older (60–75 years) adults from the LEMON study. We found significant difference in FI and CF between the older and younger adults, regardless of gender. However, neither crystalized intelligence nor WM differed between the two age groups. We used Random forests to determine whether brain volumes were predictive of cognitive measures and to identify brain subregions best correlated with each cognitive measure (Figure 2). We found group‐specific significant correlations for young adults between nucleus accumbens and fluid intelligence (*R* = −0.24, *p* = .004) and for older adults between nucleus accumbens and crystallized intelligence (*R* = −0.325, *p* = .024). Significant correlations were also found between pallidum and crystallized intelligence for young adults (*R* = −0.194, *p* = .019) and between putamen and crystallized intelligence for older adults (*R* = −0.362, *p* = .011). There were significant differences between the young adult group and the older adult group for the correlation coefficients between crystallized intelligence and putamen (*R*_YA_ = 0.041, *R*_OA_ = −0.362, *p* = .014) and between crystallized intelligence and nucleus accumbens (*R*_YA_ = 0.018, *R*_OA_ = −0.325, *p* = .038).

### 3.1 Prediction performance of three data sets

**FIGURE 3:**
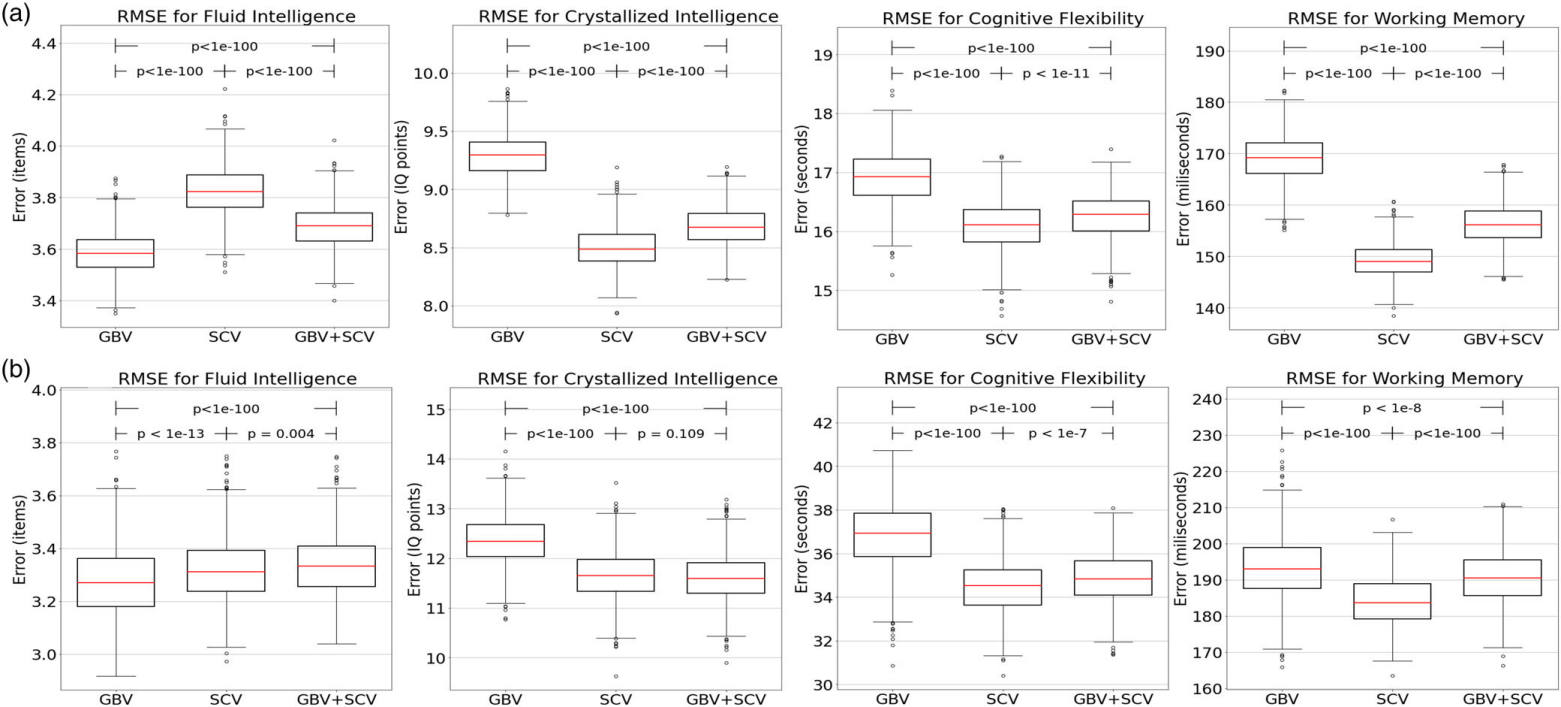
Box plots of RMSE and post hoc adjusted *p* values for correlations of cognitive measures with baseline data set of normalized global brain volumes (GBVs), data set of normalized subcortical brain volumes (SCVs), and the combined data set (GBV + SCV) in (a) young adults and (b) older adults. Red bold line: Median. Boxes: Interquartile ranges (IQRs). Whiskers: Max and min values not exceeding each 1.5 IQR. Circles: Outer values. RMSE, root mean squared error.

Three data sets were analyzed. The baseline data set included volumes of gray matter, cortical white matter, and CSF normalized to eTIV. The second data set included eTIV‐normalized volumes of seven subcortical regions. The combined data set was the union of these two data sets. We used RMSE as the primary measure to quantify the prediction accuracy of the random forest analysis. The RMSEs averaged over 1000 simulations are presented for young and older adult groups in Table 3 and Table 4, respectively. Boxplots of RMSEs for each cognitive measure and each data set are shown for younger and older adults in Figure 3. The baseline data set was the best performing data set only for the fluid intelligence measure for both younger adult (0.358 ± 0.08) and older adult (0.327 ± 0.13) groups; for other cognitive measures, the subcortical volume data set always performed better than the baseline data set. Crystallized intelligence was best predicted by the subcortical volumes data set in young adults (8.50 ± 0.18). In older adults, crystallized intelligence was best predicted by the combined data set (11.61 ± 0.47), although this was not significantly different from the performance of the subcortical volumes data set (11.66 ± 0.50). Cognitive flexibility and working memory were both best predicted by the subcortical volumes for both younger (149.16 ± 3.28) and older adults (183.03 ± 7.06). As a secondary measure in quantifying the prediction accuracy, Pearson correlation coefficients were also determined (Figure S1).

**TABLE 3:** RMSE values of cognitive measure predictions for all data sets in young adults^a^

Data sets	Fluid intelligence (items)	Crystallized intelligence (IQ points)	Cognitive flexibility (s)	Working memory (ms)
Global brain volumes	3.58 (0.08)	9.29 (0.18)	16.91 (0.44)	169.14 (4.55)
Subcortical volumes	3.82 (0.09)	8.50 (0.18)	16.08 (0.42)	149.16 (3.28)
Combined volumes	3.69 (0.08)	8.68 (0.17)	16.25 (0.39)	156.25 (3.84)

*Note*: Significant values are shaded blue.

Abbreviations: IQ, intelligence quotient; RMSE, root mean squared error.

^a^ Average RMSE (standard deviation in parentheses) over 1000 simulations.

**TABLE 4:** RMSE values of cognitive measure predictions for all data sets in older adults^a^

Data sets	Fluid intelligence (items)	Crystallized intelligence (IQ points)	Cognitive flexibility (s)	Working memory (ms)
Global brain volumes	3.27 (0.13)	12.35 (0.49)	36.80 (1.55)	193.36 (8.66)
Subcortical volumes	3.32 (0.12)	11.66 (0.50)	34.48 (1.21)	184.03 (7.06)
Combined volumes	3.33 (0.11)	11.61 (0.47)	34.87 (1.17)	190.71 (7.05)

*Note*: Significant values are shaded blue.

Abbreviations: IQ, intelligence quotient; RMSE, root mean squared error.

^a^ Average RMSE (standard deviation in parentheses) over 1000 simulations.

### 3.2 Feature importance analysis identifies brain subregions predictive of cognitive ability

**FIGURE 4:**
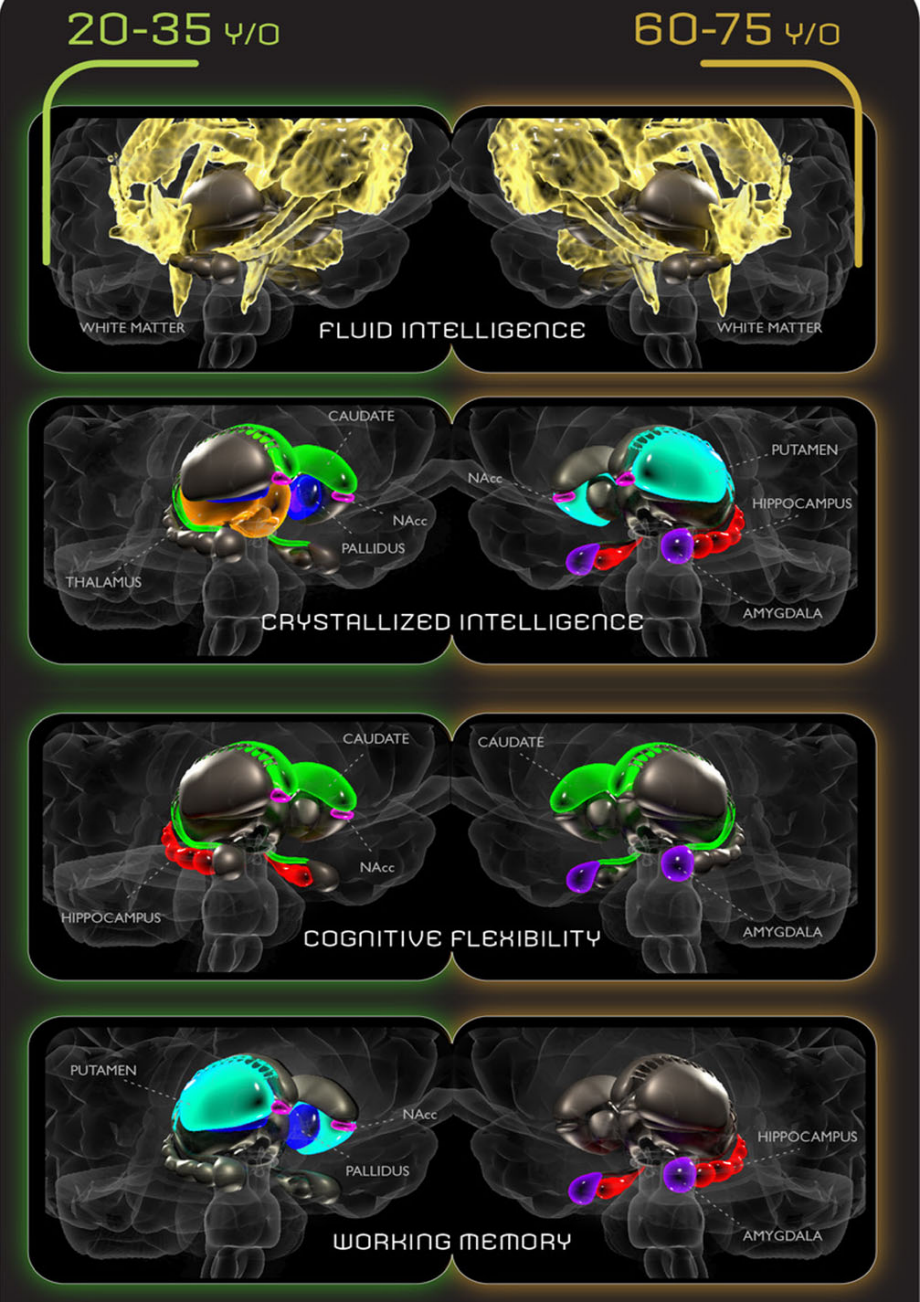
Feature importance for each behavior measure illustrated on brain hemispheres for young adults (left) and older adults (right). Structures of importance are highlighted: White matter (golden), caudate (green), putamen (turquoise), amygdala (purple), hippocampus (red), palladium (blue), nucleus accumbens (pink), and thalamus (beige).

To gain an understanding of the global and subcortical brain structures that drive the predictions of cognitive ability, we inspected the feature importance of our best‐performing data sets for each cognitive measure. Subcortical structures correlated with each behavior measure are presented in Figure 4. Tables 5 and 6 summarize feature importance frequencies for younger and older adults, respectively. Corresponding feature importance plots are shown in Figures S2 and S3. Cortical white matter was the most important feature in predicting fluid intelligence for both young and older adults. For predicting crystallized intelligence in young adults, caudate (0.154), thalamus (0.144), nucleus accumbens (0.154), and pallidum (0.211) were the most important features, whereas the most important features for older adults were putamen (0.151), amygdala (0.181), nucleus accumbens (0.150), and the hippocampus (0.202). Caudate (0.166), nucleus accumbens (0.221), and hippocampus (0.155) were the most predictive features of cognitive flexibility in young adults, whereas caudate (0.174) and amygdala (0.203) were the most predictive for the older adults. For prediction of working memory in young adults, putamen (0.153), pallidum (0.193), and nucleus accumbens (0.161) were the most important features, whereas for the older adults, amygdala (0.168) and hippocampus (0.252) were the most important.

**TABLE 5:** Average feature importance frequencies for selected brain regions in young adults^a^

Region	Fluid intelligence	Crystallized intelligence	Cognitive flexibility	Working memory
tGMV	0.290	–	–	–
cWM	0.395	–	–	–
CSF	0.315	–	–	–
Caudate	–	0.154	0.166	0.090
Putamen	–	0.109	0.119	0.153
Thalamus	–	0.144	0.120	0.131
Pallidum	–	0.211	0.115	0.193
Amygdala	–	0.118	0.104	0.142
Nucleus accumbens	–	0.154	0.221	0.161
Hippocampus	–	0.110	0.155	0.131

*Note*: Statistically significant values after a one‐sample *t*‐test are indicated by blue shading.

Abbreviations: CSF, cerebrospinal fluid; cWM, combined white matter volume; tGMV, total gray matter volume.

^a^ Equal importance threshold for fluid intelligence is 1/3 (0.333); thresholds for crystallized intelligence, cognitive flexibility, and working memory are 1/7 (0.143).

**TABLE 6:** Average feature importance frequencies for selected brain regions in older adults^a^

Region	Fluid intelligence	Crystallized intelligence	Cognitive flexibility	Working memory
tGMV	0.301	–	–	–
cWM	0.395	–	–	–
CSF	0.304	–	–	–
Caudate	–	0.142	0.174	0.126
Putamen	–	0.151	0.126	0.100
Thalamus	–	0.088	0.102	0.126
Pallidum	–	0.087	0.138	0.099
Amygdala	–	0.181	0.203	0.168
Nucleus accumbens	–	0.150	0.141	0.127
Hippocampus	–	0.202	0.115	0.252

*Note*: Statistically significant values after a one‐sample *t*‐test are indicated by blue shading.

Abbreviations: CSF, cerebrospinal fluid; cWM, combined white matter volume; tGMV, total gray matter volume.

^a^ Equal importance threshold for fluid intelligence is 1/3 (0.333); thresholds for crystallized intelligence, cognitive flexibility, and working memory are 1/7 (0.143).

## 4 DISCUSSION

In this study, we used a machine‐learning approach to explore the relationships between select higher‐level cognitive measures and subcortical brain volumes determined using T1‐weighted MRI scans in healthy young and older adults obtained from the LEMON study (Babayan et al., 2019). Our aim was to identify changes in neurocognitive architecture that occur with aging. Our analysis involved four cognitive measures, fluid intelligence, crystallized intelligence, cognitive flexibility, and working memory. The volumes of certain subcortical structures were predictive of performance on these measures, but the predictive structures differed in young and older adults, possibly reflecting age‐related changes in neural processing strategies.

Fluid intelligence was best predicted for both groups by volumes of global regions of the brain (gray matter, cortical white matter, and CSF); the volume of cortical white matter had more predictive value than volumes of gray matter or CSF. Crystallized intelligence, cognitive flexibility, and working memory were better predicted by volumes of subcortical structures alone or by combining volumes of subcortical structures with those of global brain structures than by the volumes of global regions. Feature importance analysis revealed that caudate, putamen, nucleus accumbens, and hippocampus were most predictive of cognitive measures in both young and older adults. Among these structures, the nucleus accumbens was a key feature in cognitive functioning in young adults, whereas the hippocampus appears to be more central in older adults.

MRI studies have shown that the brain undergoes morphological changes during normal aging (He et al., 2021; Jiang et al., 2020). The overall brain matter volume linearly decreases with age; however, subcortical regions, especially the caudate and the hippocampus, are altered heterogeneously (Fjell et al., 2013; Good et al., 2001; Walhovd, Fjell, Reinvang, Lundervold, Dale, et al., 2005). Furthermore, there are linear declines in executive functions related to processing speed (Bashore et al., 1997) and sensory functioning (Cavazzana et al., 2018) with age, although a substantial portion of aged subjects shows only modest or no losses in cognitive functioning (Schupf et al., 2004; Wilson et al., 2002). Altered cognitive functioning in aging has been attributed to compensation, dedifferentiation, or adaptation to loss by mechanisms of plasticity (Baltes & Lindenberger, 1997; Greenwood, 2007).

Our observation that distinct subcortical profiles predict cognitive function in young and older adults suggests that processing strategies change during aging to meet executive function needs. For example, we observed that in young adults' volumes of the nucleus accumbens, a structure that is part of the reward circuitry, which mediates motivation processing and goal‐directed behavior (Hyde & Garcia‐Rill, 2019), was predictive of success in cognitive flexibility and working memory tasks. In older adults, success in the same tasks was predicted by the volume of the amygdala, which is related to emotional memory and reward learning (Piretti et al., 2020). In both groups, performance in cognitive flexibility was also predicted by volumes of the caudate nucleus, which plays an important role in motor planning and cognitive learning (Barbosa et al., 2017; Choia et al., 2020; Grahn et al., 2008; Leisman et al., 2014), and the hippocampus, a structure important in learning and memory (Anand & Dhikav, 2012). These differences in subcortical‐behavior associations between young and older adults are suggestive of a shift from reward‐ and motivation‐based decision‐making during early adult years to an emotion‐ and vigilance‐based approach in later years. Interestingly, the nucleus accumbens receives direct input from the amygdala and hippocampus and sends output to the caudate and pallidum (Nicola, 2007), reflecting a spatial proximity of these structures. Therefore, it is possible that regional, age‐related atrophy of particular subcortical regions may lead to structural and functional reorganization of networks to maintain cognitive and motor functioning. Previous neuroimaging studies provided evidence that aging brains exhibit increased prefrontal and hippocampal activity compared to younger counterparts (Grady & Craik, 2000).

Most previous studies have focused on gray matter cortical metrics (Bajaj et al., 2018; Walhovd, Fjell, Reinvang, Lundervold, Fischl, et al., 2005), although more recent studies have revealed associations of subcortical volumes and white matter connectivity with higher cognitive abilities (Burgaleta et al., 2014; Davies et al., 2018; Grazioplene et al., 2015; Zhao et al., 2019). Subcortical functions are likely to be important for higher cognitive skills, but evidence of their morphological association with cognitive abilities is sparse. To date, only a few studies have investigated the association between subcortical structures and performance on intelligence and executive function‐related tasks, and results are inconsistent (Burgaleta et al., 2014; Grazioplene et al., 2015; MacDonald et al., 2014; Rhein et al., 2014). Discrepancies might be due to sample heterogeneity, differences between administered tests, or methodological differences. Our use of a carefully selected subgroup of subjects from the LEMON cohort along with robust analyses may then reflect meaningful results, but our finding will need to be confirmed.

Our study has limitations. One is that we did not analyze children or adolescents. Since there are notable age‐related neuroanatomical changes from childhood to adulthood (Mills et al., 2016; Pujol et al., 2021), analyses of younger subjects will be necessary to provide an understanding of brain‐cognitive association across the whole lifespan. Second, the imbalances between the numbers of young adults and older adults and between the numbers of males and females within the young‐adult data set is a limitation, as our results will likely be skewed toward the majority class. We tried to overcome any imbalance by splitting the subjects into young and older adult groups and by performing gender‐stratified cross‐validation in training, but for future studies a larger and more balanced cohort should be examined. Third, in the recent ABCD‐NP challenge, 24 research teams used machine learning and deep learning techniques to predict fluid intelligence, and 19 teams developed strategies that were better than a naïve predictor. However, the study concluded that multi‐modal MRI data (i.e., diffusion and functional) was needed for accurate prediction of fluid intelligence regardless of the artificial intelligence algorithms (Pohl et al., 2019). In this study, we used only subcortical volumetric measurements, without surface, thickness, or any other multi‐modal MRI data, and, for simplicity, we only used the random forest algorithm to predict cognitive measures and to rank the regions according to their predictive power. In the future, we plan to extend our machine learning analysis to larger data sets including a wider age range of subjects and multi‐parametric MRI data.

In conclusion, our model suggests that volumes of several subcortical structures are correlated with higher‐level cognition and appear to differentially predict cognitive measures in young versus older adults. As a next step, we will focus on the interaction between subcortical regions and cortical brain regions to better understand the neurobiological factors that drive cognitive performance in young compared to older adults. Discovering associations between cognitive characteristics and noninvasive neuroimaging metrics could pinpoint the neural underpinnings of cognition and cognitive decline. Methodological advances in this area could identify objective assessment methods for typical and atypical brain function and mechanisms that could be targeted to moderate neurodegeneration.

## CONFLICT OF INTEREST

The authors declare no conflicts of interest.

## Supporting information


**FIGURE S1** Box plots of Pearson correlation coefficients and post hoc adjusted i values for correlations of cognitive measures with baseline data set of normalized global brain volumes (GBV), data set of normalized subcortical brain volumes (SCV), and the combined data set in (A) young adults and (B) older adults

**FIGURE S2** Fluid and crystallized intelligence subregion importance for (A) young adults and (B) older adults

**FIGURE S3** Cognitive flexibility and working memory subregion importance for (A) young adults and (B) older adults

## Data Availability

The data generated and analyzed in this study are available from the corresponding author upon reasonable request.
