# Supplementary figures and images for: Predictive models demonstrate age‐dependent association of subcortical volumes and cognitive measures
Source: Hum Brain Mapp. 2022 Oct 12;44(2):801–12. doi: 10.1002/hbm.26100 (PMC9842902; doi:10.1002/hbm.26100)

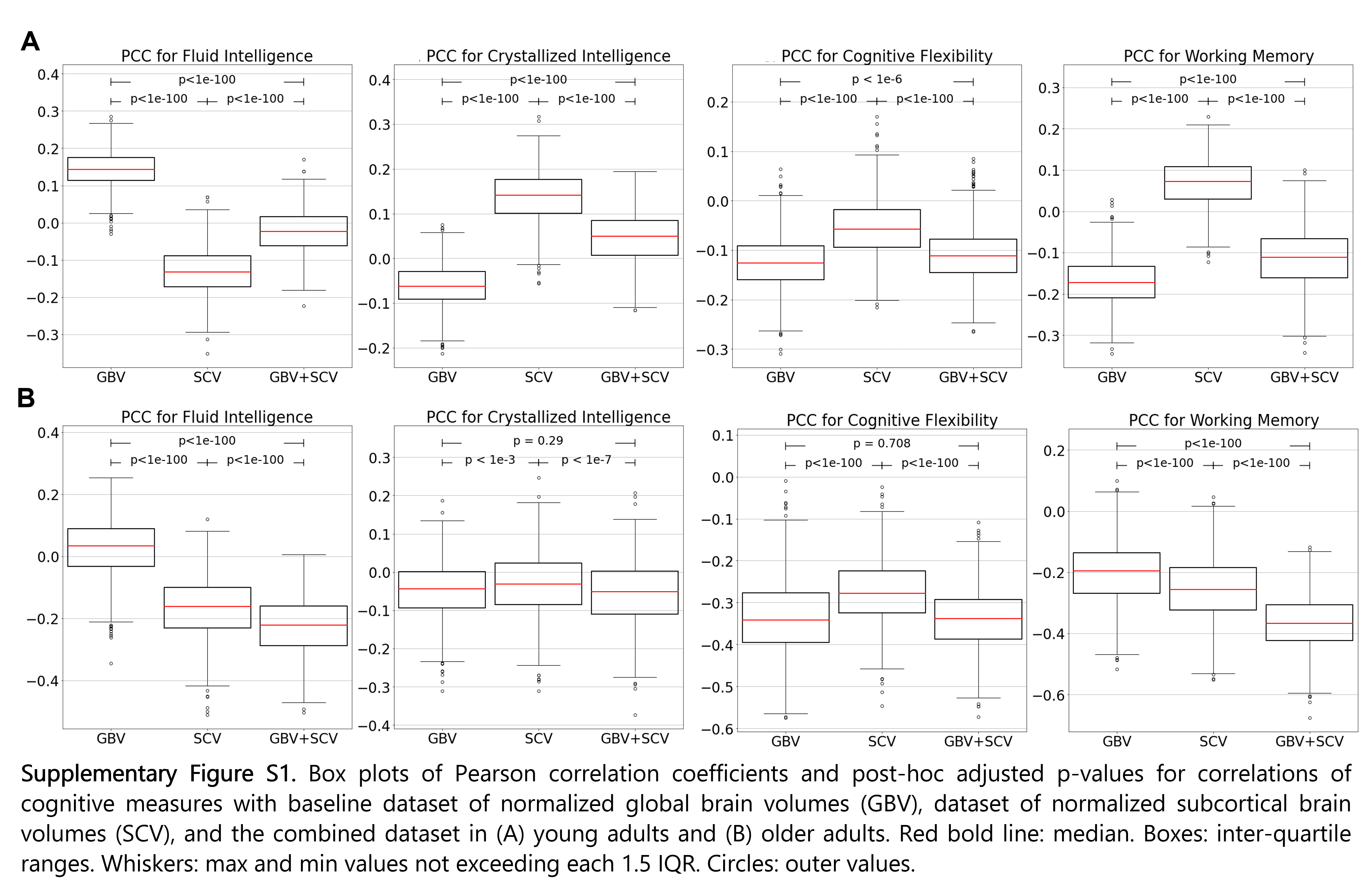

Supplement: Supplementary file 1 — FIGURE S1 Box plots of Pearson correlation coefficients and post hoc adjusted i values for correlations of cognitive measures with baseline data set of normalized global brain volumes (GBV), data set of normalized subcortical brain volumes (SCV), and the combined data set in (A) young adults and (B) older adults [file HBM-44-801-s001.tif]

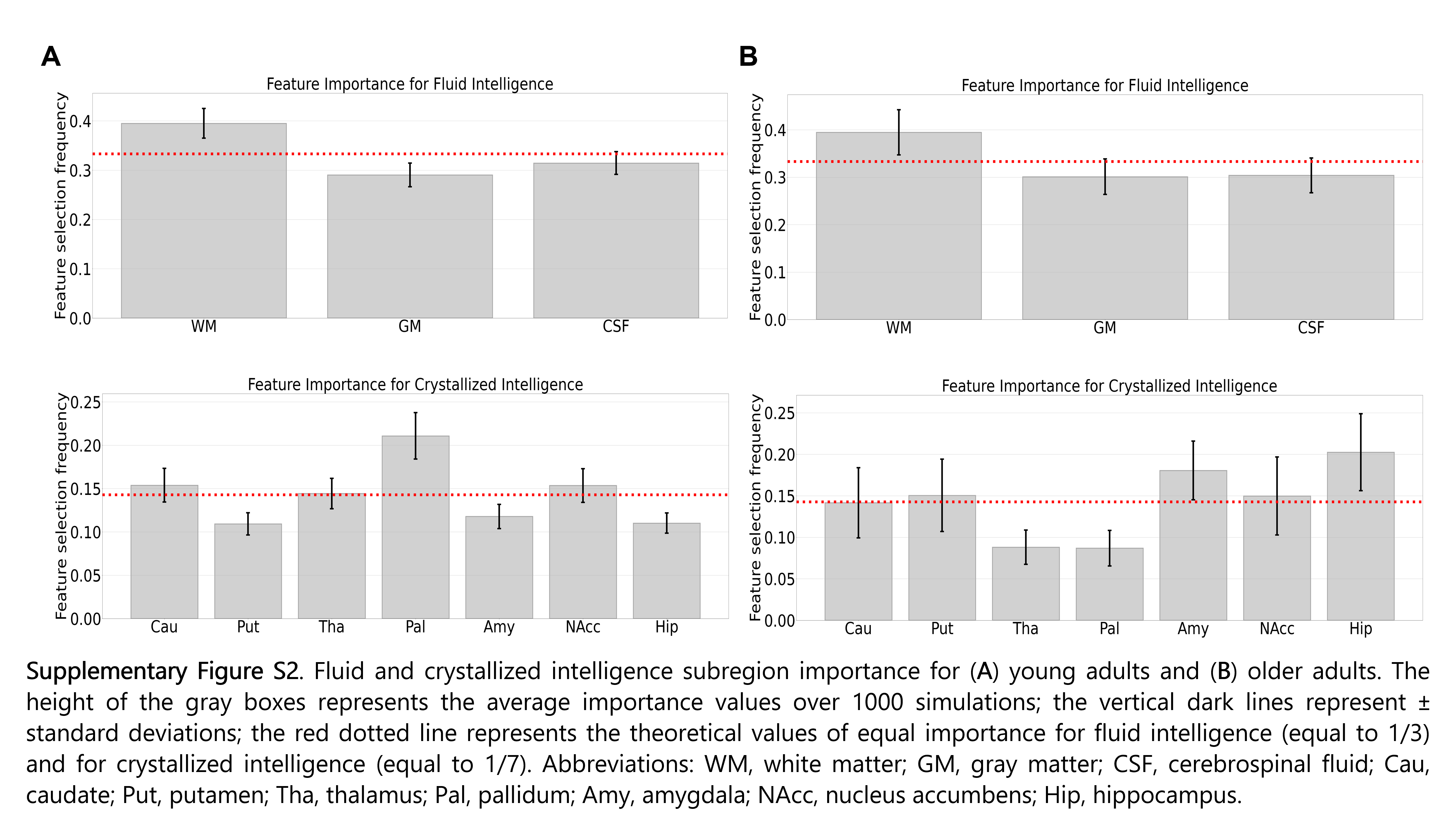

Supplement: Supplementary file 2 — FIGURE S2 Fluid and crystallized intelligence subregion importance for (A) young adults and (B) older adults [file HBM-44-801-s002.tif]

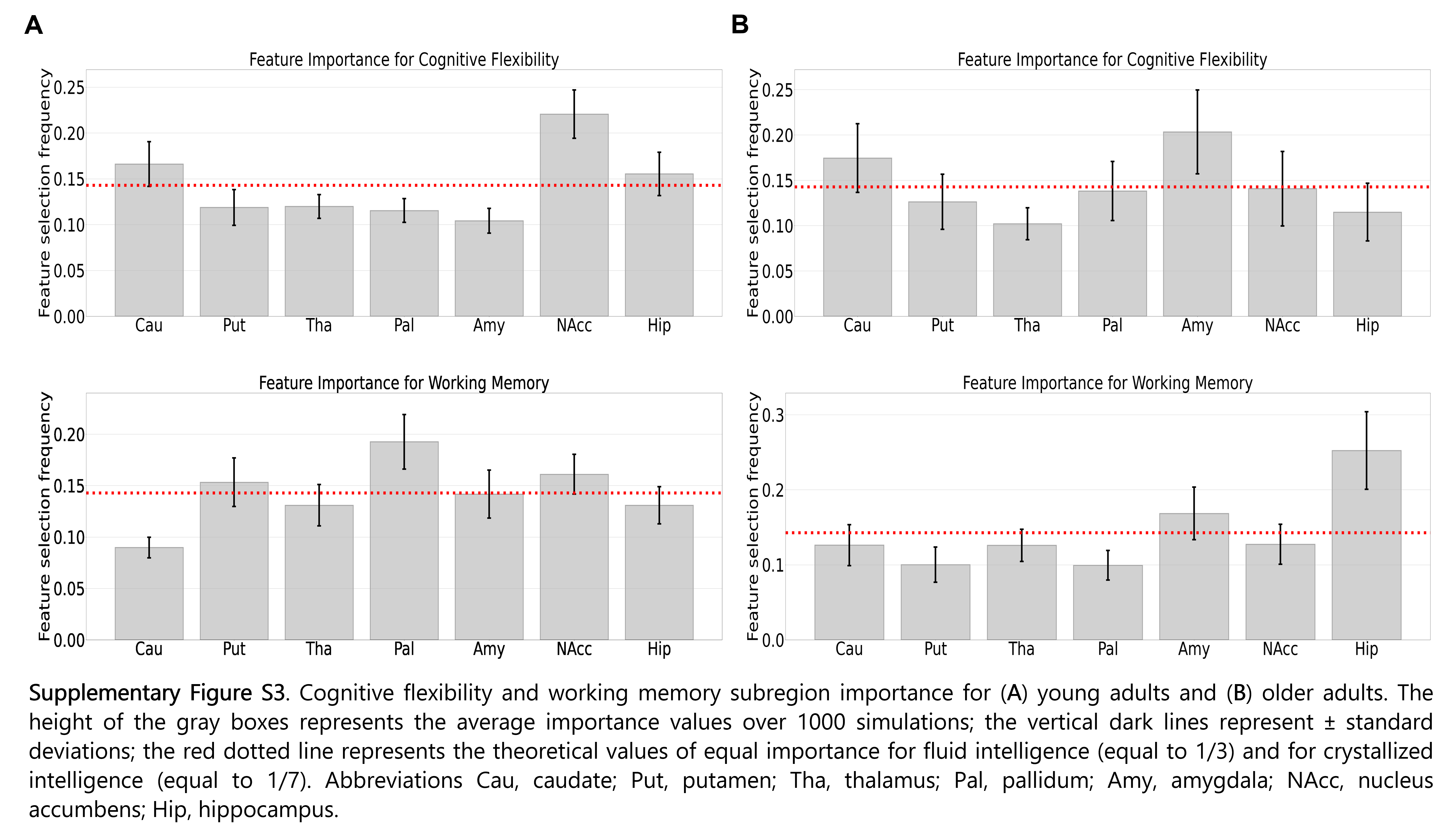

Supplement: Supplementary file 3 — FIGURE S3 Cognitive flexibility and working memory subregion importance for (A) young adults and (B) older adults [file HBM-44-801-s003.tif]
